# Evidence of stereotyped contact call use in narwhal (*Monodon monoceros*) mother-calf communication

**DOI:** 10.1371/journal.pone.0254393

**Published:** 2021-08-27

**Authors:** Audra E. Ames, Susanna B. Blackwell, Outi M. Tervo, Mads Peter Heide-Jørgensen

**Affiliations:** 1 Fundación Oceanogràfic de la Comunitat Valenciana, Valencia, Spain; 2 Greeneridge Sciences, Inc., Santa Barbara, California, United States of America; 3 Department of Birds and Mammals, Greenland Institute of Natural Resources, Copenhagen, Denmark; 4 Department of Birds and Mammals, Greenland Institute of Natural Resources, Nuuk, Greenland; Universite Paris-Sud, FRANCE

## Abstract

Narwhals (*Monodon monoceros*) are gregarious toothed whales that strictly reside in the high Arctic. They produce a broad range of signal types; however, studies of narwhal vocalizations have been mostly descriptive of the sounds available in the species’ overall repertoire. Little is known regarding the functions of highly stereotyped mixed calls (i.e., biphonations with both sound elements produced simultaneously), although preliminary evidence has suggested that such vocalizations are individually distinctive and function as contact calls. Here we provide evidence that supports this notion in narwhal mother-calf communication. A female narwhal was tagged as part of larger studies on the life history and acoustic behavior of narwhals. At the time of tagging, it became apparent that the female had a calf, which remained close by during the tagging event. We found that the narwhal mother produced a distinct, highly stereotyped mixed call when separated from her calf and immediately after release from capture, which we interpret as preliminary evidence for contact call use between the mother and her calf. The mother’s mixed call production occurred continually over the 4.2 day recording period in addition to a second prominent but different stereotyped mixed call which we believe belonged to the narwhal calf. Thus, narwhal mothers produce highly stereotyped contact calls when separated from their calves, and it appears that narwhal calves similarly produce distinct, stereotyped mixed calls which we hypothesize also contribute to maintaining mother-calf contact. We compared this behavior to the acoustic behavior of two other adult females without calves, but also each with a unique, stereotyped call type. While we provide additional support for individual distinctiveness across narwhal contact calls, more research is necessary to determine whether these calls are vocal signatures which broadcast identity.

## Introduction

Narwhals (*Monodon monoceros*) are deep-diving, medium-sized toothed whales endemic to the Arctic waters of Greenland and Northeastern Canada [e.g., 1, 2]. Like other toothed whales, narwhals are a vociferous species, using echolocation to navigate and forage within their environment [3–8] and produce a diverse repertoire of sound types for communication with conspecifics [3, 6, 9–12]. Sound types are classified into general groups of signals which share similar characteristics [13]. Those occurring in the narwhal vocal repertoire include tonal and pulsed sounds [3, 6, 9–12] and sounds which combine both tonal and pulsed components [10, 12].

Tonal sounds, including whistles, are narrowband and frequency modulated. In narwhals, tonal sounds are recorded more infrequently than pulsed sounds [3, 5–7, 10, 12] but may broadcast group or contextual information [10, 11]. Pulsed sounds are discrete packets of broadband non-echolocation pulses often defined by pulse repetition rate, or the number of pulses per second [e.g., 3, 9, 10, 12]. Narwhals also produce tonal and pulsed sounds simultaneously, resulting in biphonations with an underlying pulsed component overlapped by a tonal component. Previously, these have been referred to as “combined tonal/pulsed signals” [10], but will be referred to here as mixed calls [12–15]. In other odontocete species (e.g., belugas, *Delphinapterus leucas* [13, 16, 17]) mixed calls can also refer to signals with two overlapping pulsed elements of varying repetition rates, although such signals have not yet been reported for narwhals.

The functions of these different sounds in narwhal communication are virtually unknown, as research has been limited by the unique challenges of studying a species that strictly resides in remote high Arctic habitats and that does not survive in managed care [11, 18]. As a result, few studies exist on the communication of this species. Of these existing studies, most have relied on hydrophone deployments among herds of animals [3, 6, 9, 11]; hence, even less is known regarding the individual vocal repertoires of narwhals. Ford and Fisher [3] reported long series of highly stereotyped pulsed signals which they speculated were specific to the repertoires of individual whales. Shapiro [10] provided preliminary empirical support for this notion when two tagged whales each produced a distinct, highly stereotyped whistle and mixed call set that was dissimilar to that of the other tagged whale. It was hypothesized that such vocalizations acted as contact calls, similar to the signature whistles of bottlenose dolphins (*Tursiops truncatus*) [10], which are known to be individually specific [e.g., 19–21] and used to broadcast identity [e.g., 20, 22–24]. During separation contexts, belugas also produce contact calls which are distinct, highly stereotyped, and mixed [14, 17, 25] that may also broadcast individual or group identity [13–15, 17, 26]. Narwhals are closely related to belugas as the only two monodontid taxa, so it is conceivable that these species would have evolved to produce similar sounds in similar contexts. However, further research is necessary to elucidate the function of highly stereotyped narwhal mixed calls; namely, whether they 1) are contact calls, 2) are individually specific, and 3) act as individual signatures (i.e., broadcast identity information [27]).

Here we provide evidence that these distinct, stereotyped mixed calls function as contact calls and further support for the hypothesis that narwhals produce individually specific call types (i.e., highly stereotyped sounds that may be unique to the repertoires of one or more individuals [13]) that may broadcast identity information. A female narwhal with a calf was tagged as part of larger studies on the life history and acoustic behavior of narwhals [8, 28], thus presenting a unique opportunity to explore aspects of narwhal mother-calf communication. We show that the narwhal mother produced a distinct, highly stereotyped mixed call type when separated from her calf and immediately upon release from capture, which is evidence that this signal functioned as the female’s contact call. We also provide what is possibly the first record of narwhal calf vocalizations as we indicate that a second distinct mixed call type belonged to the narwhal calf. Based on the vocal behavior surrounding the production of this second mixed call type, we hypothesize that this signal is the calf’s own contact call. Thus, narwhal mother-calf communication appears to include individually distinct call types that are likely used to maintain contact within the mother-calf dyad, and may facilitate recognition between individuals.

## Methods

### Subjects and tagging

The female narwhal (from here on referred to as “Eistla”, 2016MM3) and calf belong to an isolated population of East Greenland narwhals that reside in the Scoresby Sound fjord system during the summer. Eistla was live-captured and tagged from a field station (Hjørnedal) in this fjord system during August 2016. All narwhal captures during the larger studies were accomplished using set nets (40 or 80 m length, 5–8 m deep) in collaboration with local Inuit hunters [29]. Further details of the study area, capture event, and tagging are specified in Blackwell et al. [8] and Heide-Jørgensen et al. [28]. The team handling Eistla during her capture event noticed that she was accompanied by a calf, as one remained near the site of capture and immediately joined Eistla upon her release. The calf appeared to be a calf of the year, estimated to be no more than five months of age.

During the capture event, Eistla was instrumented with an Acousonde™ acoustic and orientation tag (Acoustimetrics, Greeneridge Sciences, Inc., Santa Barbara, CA). The Acousonde was attached to Eistla’s skin via suction cups on her rear half, adjacent to her dorsal ridge. To extend the longevity of the attachment, two 1-mm nylon lines were threaded through the top of the dorsal ridge. The Acousonde was held to the lines with magnesium corrodible links, which allowed for approximately four days of acoustic recordings (records were available from the time of instrumentation on 24 Aug. at 13:07 until 28 Aug. at 19:47) before the tag released. After Eistla’s release, Eistla and the calf rejoined and left the tagging site together. No other visual observations of the mother and calf occurred. Additionally, for comparative purposes, we discuss signal production in two other females of the same population that were tagged in earlier years.

### Permitting

The government of Greenland granted permission for the capturing, handling, and tagging of narwhals (Case ID 2010–035453, document number 429 926), as well as access and permits to use land facilities in the Scoresby Sound fjord complex. Project approval was granted by the Institutional Animal Care and Use Committee (IACUC) of the University of Copenhagen (17 June 2015).

### Acoustic recordings

During recording, the Acousonde alternated between two acoustic sampling channels (one low-frequency and one high-frequency) in order to preserve the tag’s battery-life and storage. The low-frequency (LF) acoustic channel included an HTI-96-MIN hydrophone with a nominal sensitivity of -201 dB re 1 V μPa^-1^, sampling at a rate of 25.8 kHz. The high-frequency (HF) acoustic channel included an HTI-99-HF hydrophone with a nominal sensitivity of -204 dB re 1 V μPa^-1^, sampling at a rate of 154.9 kHz. The tag alternated between the two hydrophones, sampling 8 min on the LF channel followed by 7 min on the HF channel. All acoustic data were recorded with 16-bit resolution (see [8] for further detail on the Acousonde sampling regimens and hydrophone specifics).

### Sound analyses

Acoustic recordings were manually examined by two authors (SBB and OMT) using MTViewer (a custom-written program for analysis of Acousonde data, W.C. Burgess, pers. com.) to determine the time of occurrence of any non-echolocation signals within the records [8]. AEA then reviewed and analyzed these signals, classifying vocalizations based on existing definitions from the literature [e.g., 10, 11, 13–15, 25, 26]. Signal classification was completed in Raven Pro 1.5 (Cornell Lab of Ornithology) using a Hann window (size: 512) with a Discrete Fourier Transform (DFT) size of 512 samples and 256 hop size.

A call type was defined if a clearly distinct, highly stereotyped call appeared at least five times within the acoustic records across a minimum of two separate vocal events [13, 14]. A vocal event was defined as a single appearance of one of the following vocal behaviors, which we used to explore narwhal communication surrounding the production of specific call types. We sought to determine if call types appeared more consistently 1) in a series, 2) in a bout, 3) independent of other vocalizations, 4) as a call, or 5) as a response (see Fig 5 in results). To be considered as part of a ***series***, a call type emission had to occur within 10 seconds of at least one additional emission of the same call type. This criterion was based on the SIGnature IDentification (SIGID) method presented by Janik and colleagues [30] as a conservative means to identify signature whistles among free-ranging bottlenose dolphins. We adapted this method as a proxy for identifying possible narwhal contact calls. A ***bout*** was defined as an emission of a call type within 10 seconds of different call or sound types (excluding echolocation), presumed to belong to the same whale based on the similar energy and bandwidth of each signal within the bout on a spectrogram [12, 13]. A ***call*** was an emission, i.e., a signal initiating communication with another whale, if it was followed by a different signal of varying energy and bandwidth that overlapped the call or was within a second of the call. A ***response*** was a signal that occurred overlapping or within one second of an initiating call. Additionally, if a call or response occurred within a series or bout, the other signals within the same series or bout were also considered to be calls or responses. Signals that occurred without the presence of other signals within 10 seconds prior to or following the signal were considered to be single, or independent vocalizations.

During the tagging procedure, while flanked on each side by members of the tagging team, Eistla produced several loud identical vocalizations recorded by the Acousonde, which was not yet attached but held underwater within approximately two meters to the right of Eistla’s head. These signals represented a possible call type within Eistla’s repertoire to which we could match other signals that were recorded following her release. Later signals that matched these stereotyped vocalizations that had high signal-to-noise ratios and maintained consistent energy and bandwidth within and between vocal events [12, 13] were also presumed to belong to Eistla. Consequently, signals that were variable in energy and bandwidth during vocal events, or that overlapped Eistla’s known call type, were considered to belong to a different whale.

Sounds that did not meet call type criteria were cataloged according to the sound classification groups identified for narwhal vocal repertoires, i.e., tonal, pulsed, and mixed calls [e.g., 3, 6, 9–12]. These sounds were then further classified based on whether the signal was likely produced by Eistla or another whale. As with Eistla’s stereotyped vocalizations, non-distinct sounds that had high signal-to-noise ratios and maintained consistent energy and bandwidth throughout the recordings were considered to belong to Eistla. Sounds that appeared with variable or faint energy were considered to belong to another whale, including possible calf sounds as well as contact calls of other whales which were not produced enough in the record to meet our call type criteria. Calls were conservatively identified as belonging to the calf based on signals which were produced throughout the recordings that were tremulous—a quality associated with odontocete calf vocalizations (e.g., belugas [13, 14]; bottlenose dolphins [31, 32]). Signals that did not meet the criteria of clearly belonging to Eistla, the calf, or another whale were classified as unknown. These were signals with energy that did not allow for clear identification of the signaling whale or signals that were overlapped with noise, making them unanalyzable.

Finally, we manually inspected 612 vocalizations obtained from the LF and HF acoustic recordings of two other female whales (Freya, 2013MM3, and Thora, 2014MM6 [8]). These females were not known to have calves at the time of capture, so we used these recordings to determine if the acoustic behavior present in Eistla’s records was similar to that of females without calves. Signals in the records of these females were classified using the same method for identifying call types and non-distinct sound types as were produced in Eistla’s record. Signal energy and bandwidth were also used to determine the identity of the signaling animal within the acoustic records of these additional females.

### Parameter extraction

Parameters were extracted from distinct call types within each female’s record for further analyses. Only calls produced in HF recordings that did not overlap with other signals or noise and had a high signal-to-noise ratio (minimum signal-to-noise ratio of 20 dB) were considered for parameter extraction. Additionally, signals used for parameter extraction were limited to those produced when tagged whales were at < 20 m depth, as 1) 18 m was the maximum recording depth of possible calf vocalizations that met parameter extraction criteria and 2) increased hydrostatic pressure has been shown to affect parameters of acoustic energy distribution in beluga signals [33]. The depth at which each signal was recorded was determined prior to analyses for this study (for details on depth sampling and analyses, see [8]). It should be noted that the call type described for Freya below was never produced < 20 m, so we extracted parameters from the few available examples in her HF record, regardless of their depth.

Signal duration (s), pulse repetition rate (PRR, the number of pulses/s), peak frequency (the peak power of a sound on the power spectrum, in kHz), and number of tonal or pulsed elements overlapping the underlying pulsed component were extracted for an entire signal. For peak frequency measurement, we separated Eistla’s vocalizations produced before and after her release due to the difference in Acousonde position relative to the sound source. Inter-signal intervals (ISI) were also extracted between signals in series and bouts to measure similarity in temporal production to other known contact calls (dolphin signature whistles [30]). In the case of an overlapping tonal component, the dominant tonal frequency (frequency of tonal component containing the peak power), minimum and maximum (lowest and highest frequency of the dominant tonal frequency component, respectively), as well as start and end frequencies (frequency at which the dominant tonal frequency component began and ended, respectively) were also extracted. All parameters, except for PRR, were extracted in the frequency domain (Hann window size: 1024, Fourier Transform size of 1024 samples, and 512 hop size). PRR was determined by counting the pulses over the duration of a signal either by hand in Raven (Hann window size: 512 samples, Fourier Transform size of 512 samples, and 256 hop size) or by using a custom-written MATLAB script (MathWorks, Inc., Natick, MA, USA). Average PRR was then determined by dividing the signal duration by the number of pulses.

### Statistical analysis

We determined the sample size of Eistla’s calls meeting our criteria for parameter extraction and then randomly selected an equivalent sample size from Thora’s calls meeting the same criteria. Parameters from these two call types were then compared using discriminant function analyses (DFA, SPSS version 23, IBM) to demonstrate distinction between the two call types [13, 14, 17]. For Freya and the calf, samples sizes were too low for inclusion in the DFA, so parameters were used to determine descriptive statistics ($\bar{x}$ ± SD). The number of overlapping elements was treated as a discrete variable for which the median value and range in component number were determined. Range was also included for depth and ISIs. A Mann-Whitney U test (SPSS version 23) was used for statistically comparing depth of independent call type emissions to depth of all other vocal event types as inclusive group sample sizes varied slightly. Fisher’s Exact Tests (SPSS version 23) were used to compare the frequency of signal occurrences within vocal event categories since expected values were low. Lastly, it is important to note that percentages and totals reported for signal emission frequencies within vocal event categories do not equate the total number of reported signals for each call type as some signals could be considered calls/responses and also simultaneously be part of a series or bout based on our operational definitions.

## Results

A total of 500 non-echolocation sounds were apparent in Eistla’s HF and LF acoustic records (including all possible signals produced by Eistla and other whales, record duration: 102 h, 40 min), of which 148 were considered to belong to two specific call types. The remaining 352 calls did not meet call type classification criteria and thus were grouped based on broad sound categories: 116 maintained consistent energy across the recordings and were considered to belong to Eistla (pulsed and mixed calls, see S1 Table for summary of sound types produced in each female’s acoustic record) while an additional 50 were likely produced by the calf (tonal, pulsed, and mixed calls in S1 Table).

**Fig 1:**
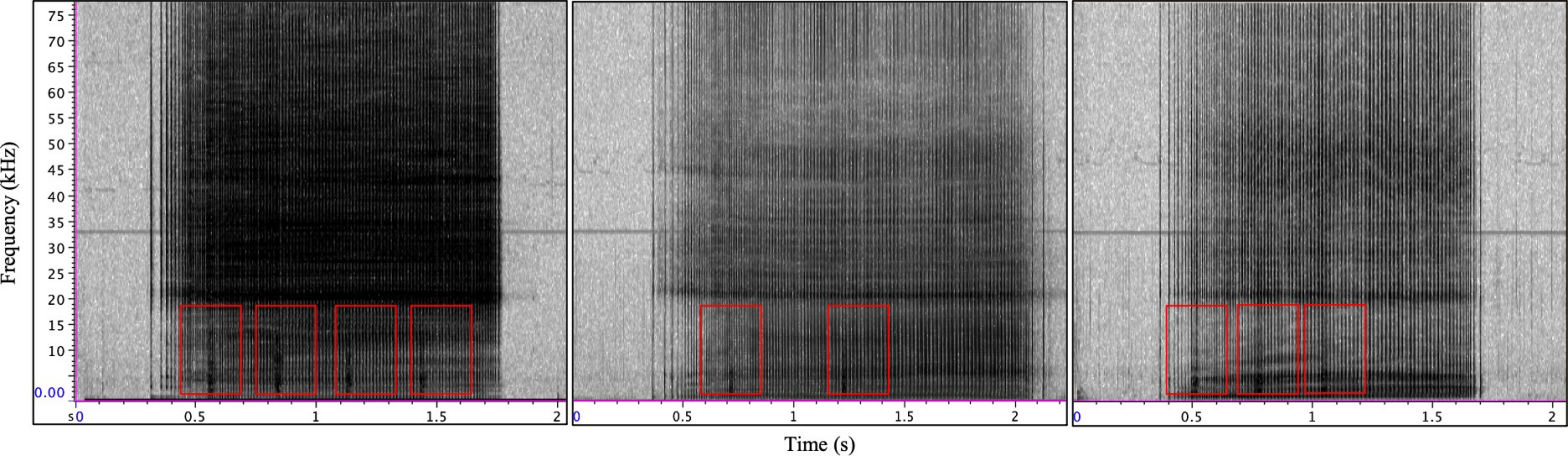
Type E examples from sample used for parameter extraction (n = 33). The far left panel is a type E call recorded during Eistla’s tagging event when the Acousonde was held underwater near her head. The center and right panels are type E examples from the remainder of the recording period following Eistla’s release. Red boxes indicate type E overlapping pulsed components. Spectrogram parameters: DFT size 512, 256 sample hop size, and Hann window 512 samples.

**Fig 2:**
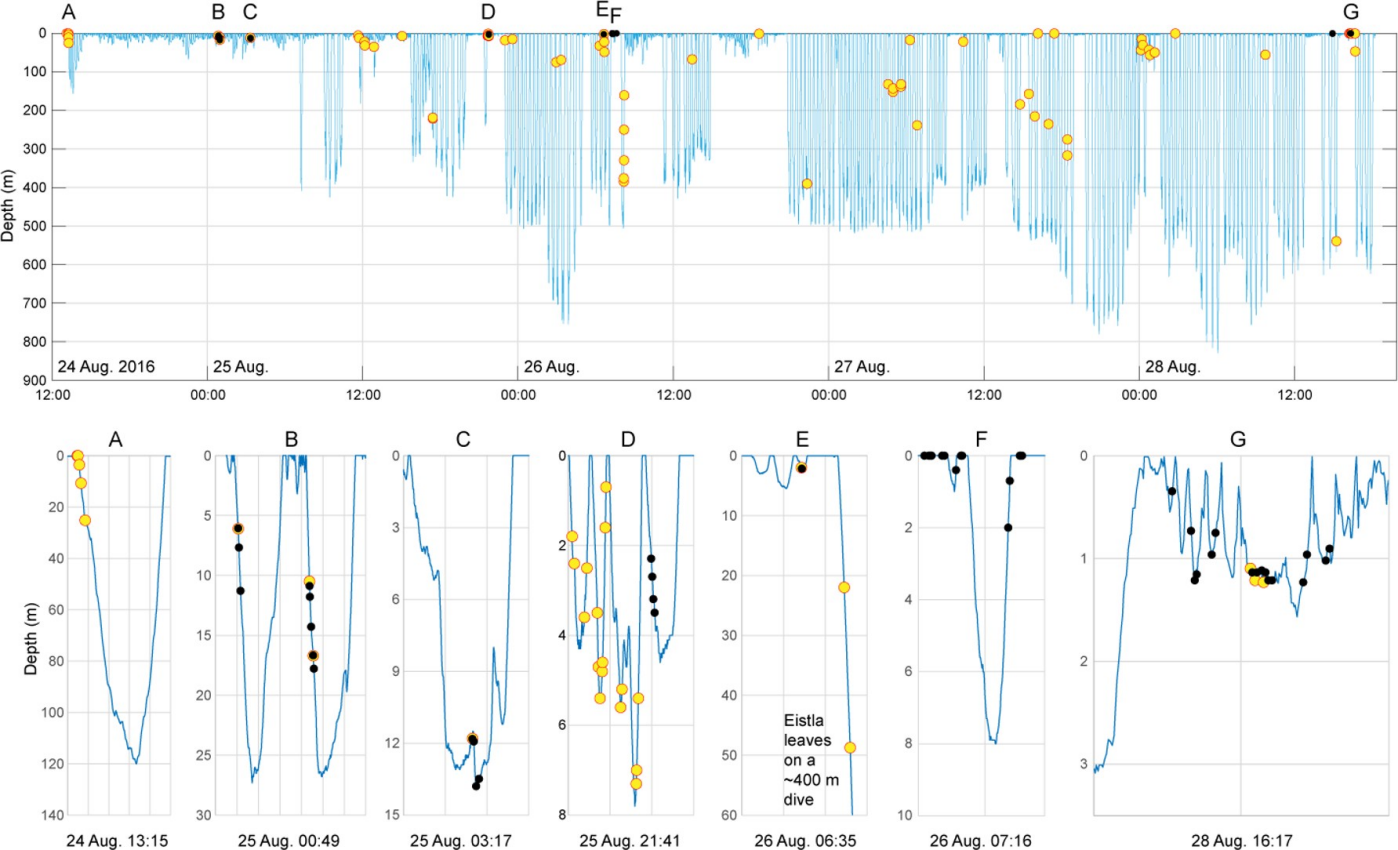
Eistla’s time-depth dive record overlaid with occurrences of type E and C signals. The top panel shows Eistla’s entire Acousonde record. Yellow dots represent type E calls, while black dots represent type C. In subpanels A-G, the x-axis is labeled with the start time and date of each panel, and each vertical interval represents two minutes. Panel A: Type E calls produced immediately following Eistla’s release. Panels B-C: A call and response exchange between type E and C. Panel D: Eistla returns from a ~230 m dive and five type E series (16 calls in the series) are produced while she lingers near the surface followed by one type C series (4 calls in the series). Panel E: One type E call followed by a type C response with type E signals continuing as Eistla leaves on a ~400 m dive. Panel F: Five type C series (14 calls in the series). Panel G: One type C response to a possible other whale (not featured in figure) followed by a type C series, two type C calls with a pulsed sound response (not featured in figured), a call and response exchange between type E and type C, and two type C series. Note that some signals are overlapped and therefore not all signals are clearly visible in the figure.

While Eistla was held by the tagging team, she produced a clearly stereotyped, broadband mixed call consisting of two overlapping pulsed components (Fig 1), five times in a row. She produced this call another five times within the first two minutes after her release (Fig 2A), and thus we determined this to be a clear call type, labeled as call type “E”. This call type had a visibly consistent PRR in the underlying pulsed component, while the overlapping pulsed components had the aural quality of brief pulsed “chirps” or occasionally, pulsed tones. The type E call was recorded every day over the five-day recording period (n = 94 total occurrences, 18.8% of total sounds identified in Eistla’s record, Fig 2, S1 Table). Of the 210 total sounds believed to be produced by Eistla, the type E call constituted 44.8% of Eistla’s signal production. After her release, Eistla did not echolocate for nearly 18 h, a post-tagging “foraging silence” that is commonly observed [8, 34]. Nevertheless, she was the only whale out of the six records analyzed in [8] to vocalize immediately after release.

A total of 33 type E signals met criteria for parameter extraction. Type E duration and PRR were consistent across the sampled signals (Table 1). The peak frequency of the call type (Table 1) was lower and somewhat variable in the type E signals recorded after Eistla was released ($\bar{x}$ depth = 3.2 ± 3.5 m) compared to the signals sampled from Eistla’s tagging event (depth = 0.3 m). This would be expected considering the tag was attached to the whale’s body with suction cups, and low-frequency components of the sound production could reach the tag through tissue conduction [35]. The number of overlapping pulsed elements ranged from 0 to 7, and the ISI for all type E vocalizations in series were within 0.72 to 6.86 s (n = 25 ISIs included in analyses).

**Table 1:** Mean ± SD of parameters extracted from calls of type E (Eistla), C (calf), F (Freya), and T (Thora).

Call Type	n	Duration (s)	Pulse Repetition Rate (PRR) (pulses/s)	Peak Frequency (kHz)	Inter-signal Interval (ISI) (s)^a^	# of Overlapping Elements^b^	Dominant Tonal Frequency (kHz)	Minimum Contour Frequency (kHz)	Maximum Contour Frequency (kHz)	Start Contour Frequency (kHz)	End Contour Frequency (kHz)
**Type E**	33	1.26 ± 0.33	127.82 ± 12.04	*	3.62 ± 1.99	2	-	-	-	-	-
*Peak Frequency Before Release	5	-	-	30.25 ± 6.30	-	-	-	-	-	-	-
*Peak Frequency After Release	28	-	-	12.43 ± 9.93	-	-	-	-	-	-	-
**Type C**	7	1.39 ± 0.30	38.96 ± 6.95	15.04 ± 10.58	2.58 ± 2.19	5	8.94 ± 1.43	5.23 ± 1.23	13.83 ± 2.70	8.34 ± 4.21	5.66 ± 1.13
**Type F** ^c^	3	1.01 ± 0.16	182.42 ± 11.72	2.02 ± 0.35	-	0	-	-	-	-	-
**Type T**	33	0.65 ± 0.07	245.69 ± 32.81	19.68 ± 6.78	3.68 ± 1.90	0	-	-	-	-	-

^a^ The mean inter-signal interval (ISI) represents the ISI between signals in series only (n = 28 ISIs for type E, n = 40 ISIs for type C, n = 200 ISIs for type T, type F never appeared in a series).

^b^ Represents the median number of overlapping elements.

^c^ Depth restrictions for parameter extraction were not applied to the few calls available in Freya’s HF record.

**Fig 3:**
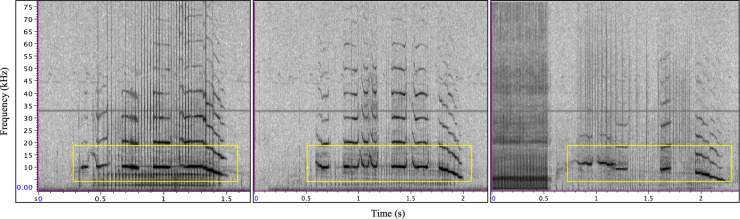
Type C examples from samples used for parameter extraction (n = 7). In all panels, note the upper frequency harmonics, indicative of a signaler facing the Acousonde. In the far right panel, note the end of a type E call (indicated by red asterisk) prior to the beginning of a type C response. Yellow boxes indicate type C overlapping tonal components. Spectrogram parameters: DFT size 512, 256 sample hop size, and Hann window 512 samples.

In addition to type E, there was another consistently produced call type (type “C”) that presumably belonged to Eistla’s calf based on its constant appearance in the acoustic records with variable energy and bandwidth within series or bouts and the presence of characteristics that have been associated with underdeveloped sounds in the odontocete literature [13, 14, 31, 32]. Additionally, the harmonic structure often contained intervals with high frequencies consistent with an animal facing the acoustic recorder [36–38]. The type C call was also a stereotyped mixed call, comprised of a pulsed and tonal component, although this call appeared to not be fully stereotyped (Fig 3). The PRR of the type C pulsed component was visibly low while the overlapping tonal component was often visually and aurally tremulous, and consisted of multiple, non-continuous concave or constant contours ending in a down-sweeping contour (Fig 3). The type C call was recorded 54 times (10.8% of all sounds) over the course of 3 different days (25, 26, and 28 August, Fig 2). The type C call constituted 51.9% of possible calf sounds (n = 104), however it should be noted that the sample size of calf sounds was highly conservative as it was limited to sounds which met criteria of tremulous sounds described for dolphin and beluga calves [13, 14, 31, 32].

A total of 7 type C signals met parameter extraction criteria (depth range of included calls: 0–18 m, $\bar{x}$ depth = 6.8 ± 7.6 m). As with type E, duration and PRR were consistent across the small sample, as was the dominant tonal frequency (Table 1). The dominant tonal frequency was also the peak frequency for 5 of the 7 calls; however, the overall peak frequency of the sampled type C emissions was skewed by the pulsed element of the remaining 2 sampled calls. The minimum, maximum, and end contour frequencies were also consistent across the sampled calls, but the start contour frequency appeared to be less stereotyped. The number of overlapping tonal elements was in the range of 3 to 6 and the ISI of vocalizations included in series ranged from 0.78 to 9.66 s (n = 40 ISIs included in analyses).

Acoustic recordings of the two other females not known to have calves at the time of capture also contained each a distinct, stereotyped call type that were determined to belong to the two females: the type “T” call belonging to Thora and the type “F” call belonging to Freya (Table 1). Neither call type was emitted during either female’s capture and both females were silent following release (Thora for 4.4 h until the first type T call, 9.4 h until the first bout of echolocation, and Freya for 36.8 h before any sounds). Thora’s acoustic record (101.0 h from the first type T call) contained a total of 586 signals, of which the type T call was emitted in 426 occurrences (72.7%). Although we did not thoroughly investigate vocal events for call types T and F, type T was generally produced in series (n = 200 ISIs included for n = 263 calls, 61.74% of type T production).

**Fig 4:**
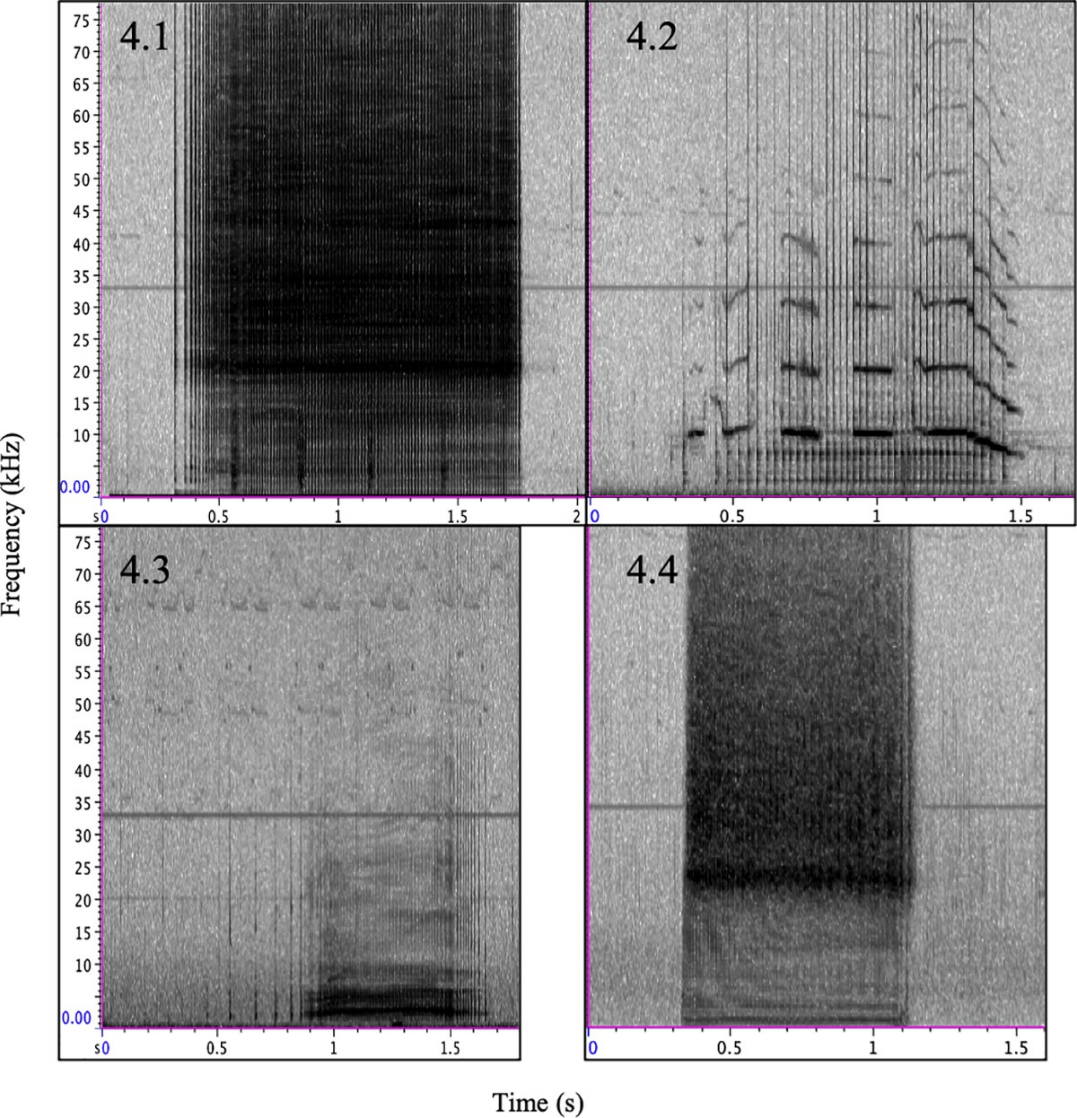
Example comparisons of type E (4.1), C (4.2), F (4.3), and T (4.4.). Spectrogram parameters: DFT size 512, 256 sample hop size, and Hann window 512 samples.

Unfortunately, Freya’s acoustic record was shorter than the other two females’ (31.4 h without initial silent period) resulting in only 26 recorded signals, of which the type F constituted 7 emissions (26.9%). No call type F emissions occurred in series. Thora’s record contained no prominent second call type. Freya’s record did contain a second signal meeting our call type criteria that was emitted an equal number of times (n = 7, 26.9%). This signal appeared to be emitted by another whale as the energy of the signal was not consistent across vocal events, but was, however, highly stereotyped, so we speculate that this signal belonged to an older whale as it lacked the tremulous quality associated with odontocete calf vocalizations (e.g., belugas [13, 14]; bottlenose dolphins [31, 32]). Fig 4 illustrates the visual differences between call types E, C, F, and T.

Based on the number of type E calls used for parameter extraction, we randomly selected 33 type T calls from Thora’s HF acoustic record (depth range of included T calls: 0.0–17.6 m, $\bar{x}$ depth = 2.5 ± 3.7 m). Parameters that were included in the DFA were call duration, PRR, and peak frequency extracted from these two call types as these were the only corresponding parameters that were extracted for both whales. The DFA classified 98.5% of calls correctly, indicating a high degree of stereotypy and difference between these two call types. Call duration (p < 0.001) and PRR (p < 0.001) were the highly discriminant parameters with peak frequency (p = 0.053) not showing discriminant ability. Consequently, we also ran a DFA with 5 type T calls and the 5 type E calls Eistla produced at the surface removed from the analyses (n = 28 calls analyzed for both call types). The DFA classified 100% of cases correctly with all parameters showing significant discriminant ability (p < 0.05).

**Fig 5:**
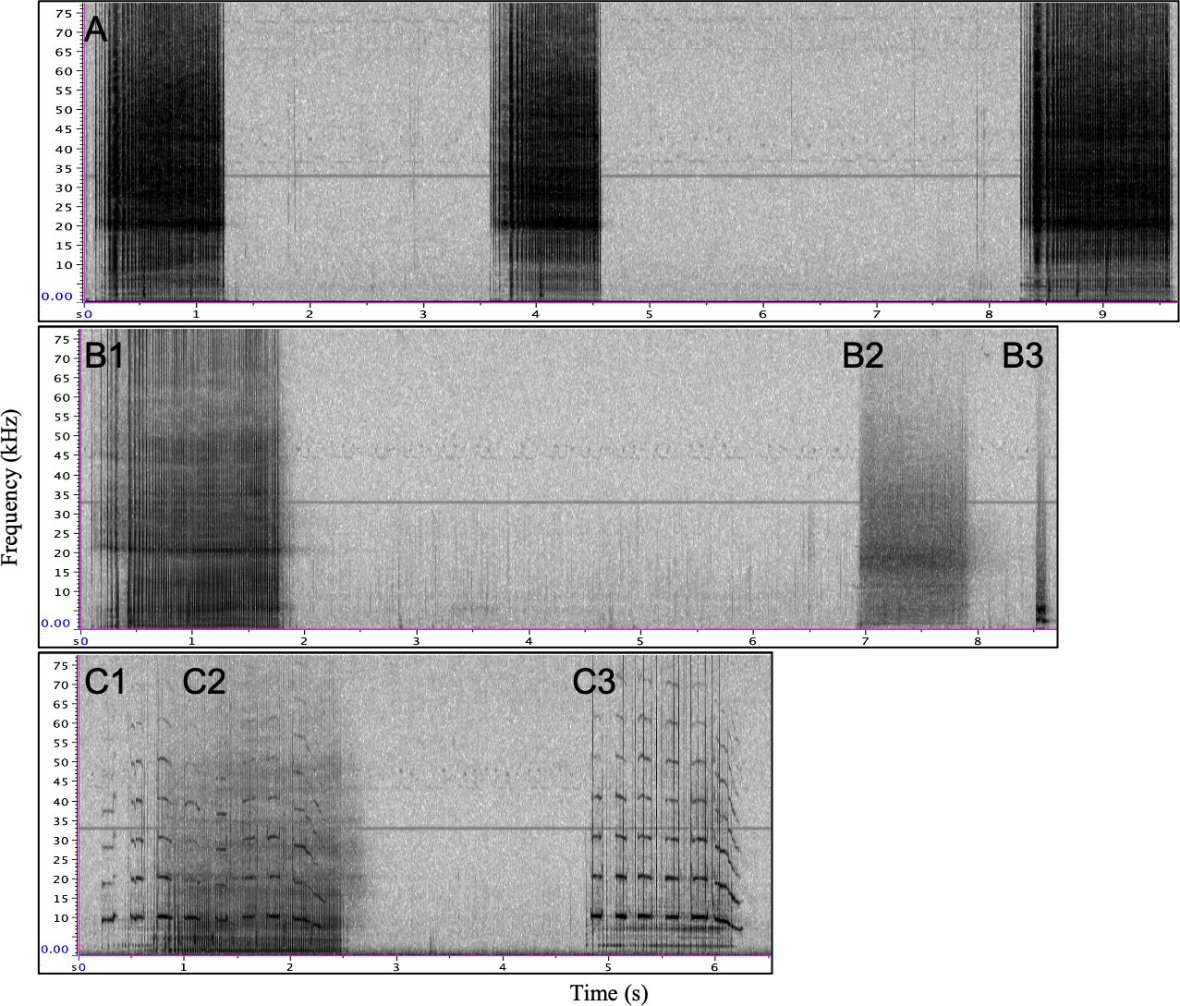
Examples of each vocal exchange type, excluding independent vocalizations. Panel A: series of type E calls; Panel B: bout containing a type E call (B1), a potential call from another whale (B2)—note the difference in energy appearance on the spectrogram when compared to B1 and B3, and a second sound type (B3) believed to belong to Eistla as it has similar energy to B1; Panel C: a call and response exchange between type C (C1 and C3) and type E (C2). Spectrogram parameters: DFT size 512, 256 sample hop size, and Hann window 512 samples.

**Fig 6:**
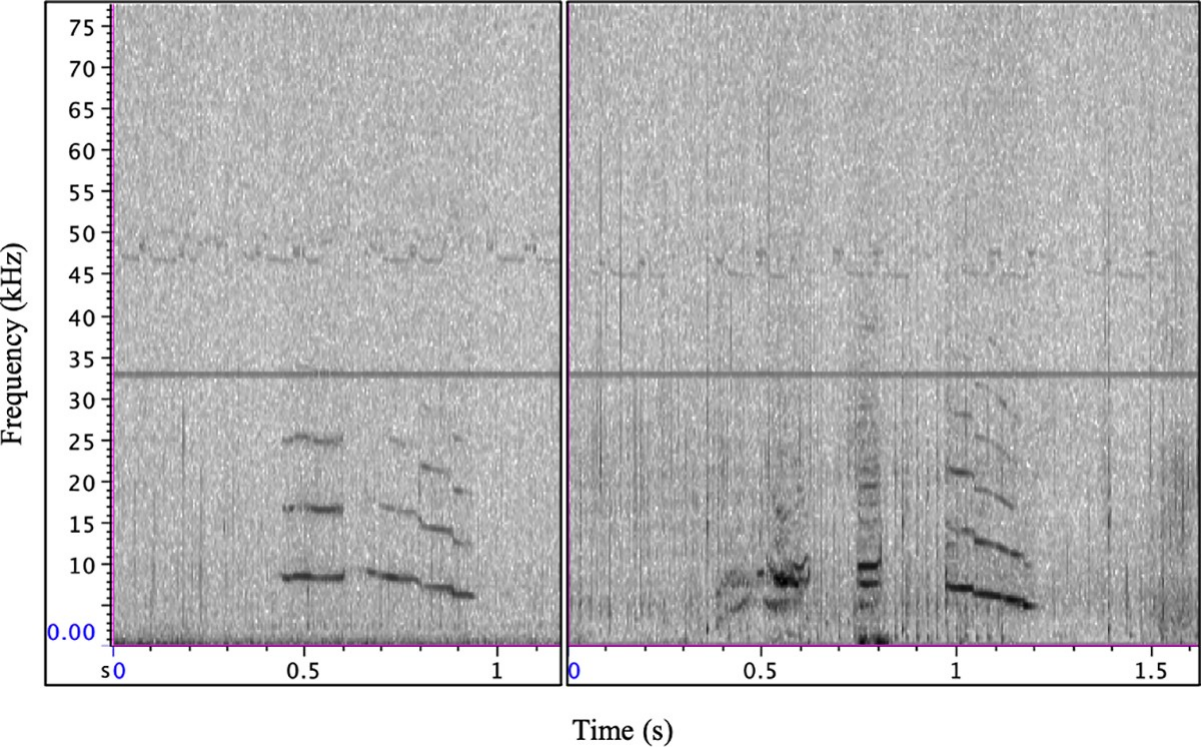
Examples of whistle contours similar to the type C call type. Note the slight deviations in stereotypy and varying spectrogram energy. Spectrogram parameters: DFT size 512, 256 sample hop size, and Hann window 512 samples.

Two-tailed Fisher’s Exact Tests showed a significant difference in the number of type E (Fisher’s Exact Test = 42.12, p < 0.001) and type C (Fisher’s Exact Test = 15.19, p = 0.009) emissions across vocal event types (see Fig 5 for example vocal event types). The type E call was produced primarily in series (n = 14 total series, n = 39 total calls in series, 41.5% of total E emissions, Fig 2A and 2D, Table 2) or independently of other sounds (excluding echolocation) or call types (n = 40, 42.6% of total E emissions). Type E was produced only 16.7% of the time in response to type C, but appeared to elicit the production of type C in 77.8% of cases in which there was an immediate vocal response from another whale (Fig 2B, 2C, 2E and 2G). The type C call was also produced primarily in series (n = 6 total series, n = 46 total calls, 85.2% of total C emissions, Fig 2D, 2F and 2G) and in response to other vocalizations (n = 20, 37.0% of total C emissions, Fig 2B, 2C, 2E and 2G) with 95% of type C responses produced in response to type E. When type C emissions elicited a vocal response from another whale (n = 7), the response signal was type E in all cases (Fig 2B and 2G). Of the two bouts containing type C occurrences, the only other signals within the bout were whistles of the same type C stereotyped contour. Both bouts occurred in the LF recordings so it is possible that these whistles were accompanied by a pulsed component that was not readily visible, for example due to the calf being at a distance from its mother where only some of the call components were detectable on the LF recording. As a result, the two “bouts” were only considered to be bouts because they could not be verified series as they contained whistles similar to the type C tonal component (i.e., contours consisting of segments that matched the type C call, Fig 6). These whistles existed often in the recordings (n = 21), and were considered as part of the calf’s vocal repertoire given the similarity in contour shape to the tonal component of type C signals. Type C was never recorded independent of any other sounds or call types.

**Table 2:** Occurrence of type E and C emissions within each vocal event type.

Call Type	Date	Total Calls	Total Responses	Total Independent	Total # in Series	Total # of Series	Total # in Bouts	Total # of Bouts
**Type E**	24-Aug	-	-	1	5	2	4	1
	25-Aug	3	1	8	22	7	-	-
	26-Aug	2	-	9	2	1	1	1
	27-Aug	-	-	11	4	2	1	1
	28-Aug	4	5	11	6	2	-	-
	**Total**	**9**	**6**	**40**	**39**	**14**	**6**	**3**
**Type C**	25-Aug	5	12	-	13	3	3	1
	26-Aug	-	1	-	14	5	3	-
	28-Aug	2	7	-	19	6	-	1
	**Total**	**7**	**20**	**-**	**46**	** 14**	**6**	**2**

While type E was recorded at varying depths (n = 94, $\bar{x}$ = 66.0 ± 111.1 m, range: 0–539 m), type C emissions were only recorded when Eistla was at or near the surface (n = 54, $\bar{x}$ = 3.1 ± 5.3 m, range: 0–18 m, Fig 2). Production of type E signals independent of other vocalizations occurred at significantly greater depths than type E production in other vocal events, such as bouts or series (Mann-Whitney U: p < 0.001). Further, production of unstereotyped sounds believed to belong to Eistla (n = 116) also occurred closer to the surface (pulsed: n = 98, $\bar{x}$ = 21.7 ± 68.1 m, range: 0–509.3 m; mixed call: n = 18, $\bar{x}$ = 22.4 ± 17.8 m, range: 0–61.6 m). Table 2 summarizes the use of each call type across the recording period.

## Discussion

### Type E and C call types

While Eistla was held for tagging, she produced the type E call type, a highly stereotyped, broadband mixed call with two overlapping pulsed components, which was emitted again shortly after her release and throughout the recording period. The type E call was prominently produced in the acoustic record when compared to other sounds we believe were produced by Eistla (S1 Table). Type E was most commonly produced either in series with emissions of the same call type, or independently of other vocalizations; however, when produced alone, type E emissions were generally produced at deeper depths (Fig 2). When type E elicited a response from another whale, the most common response was a second prominent call type, type C.

The type C call type was presumed to belong to Eistla’s calf, as this signal was also recorded throughout the tagging period but had variable energy and bandwidth (based on visual inspection of spectrograms) during acoustic events, which is consistent with an animal altering orientation to and increasing or decreasing distance from the acoustic recorder [3] in addition to having the tremulous, unstereotyped characteristics of sounds described for young animals in the odontocete literature [13, 14, 31, 32]. The harmonic structure of the type C tonal component was also often consistent with an animal facing the recorder (Fig 3; [36–38]) and this signal was sometimes produced overlapping a type E vocalization, thus providing further evidence that this signal was not produced by Eistla. The type C call type was the most prominently produced signal that we attributed to the calf (S1 Table). Type C was most commonly produced in series with other type C emissions and in response to type E vocalizations. When production of type C signals elicited a response from another whale, in all cases the response was of type E. Type E was emitted at varying depths while type C was recorded only near the surface (Fig 2), indicating that Eistla’s calf may have stayed at the surface (<18 m) during Eistla’s deep foraging dives or rejoined her near the surface during her ascent as has been shown for other deep-diving toothed whales (e.g., sperm whales, *Physeter macrocephalus* [39]).

### Evidence of contact call use in narwhal mother-calf communication

During capture and tagging, East Greenland narwhals are usually non-vocal (MP Heide-Jørgensen, pers. com.), and this is also generally the case over several hours following release as was evident with the additional two females included in our study [8]. In addition, recordings from the females without calves did not contain a second prominent call type with calf-like qualities like what we describe in this study. Thus, we hypothesize that Eistla’s production of type E during her capture and immediately upon release was to maintain contact and facilitate a reunion with her nearby calf. Given the contextual significance of Eistla’s type E production, we argue that this is the first empirical support for the existence of contact calls in narwhal mother-calf communication.

Contact calls are ubiquitous in the vocal repertoires of social, aquatic species (e.g., king penguins, *Aptenodytes patagonicus* [40]; Galapagos fur seals, *Arctocephalus galapagoensis*, and Galapagos sea lions, *Zalophus californianus wollebaeki* [41]; northern fur seals, *Callorhinus ursinus* [42]; killer whales, *Orcinus orca* [43]; belugas [17]; delphinid signature whistles, e.g., *Tursiops spp*., [44, 45]). Arguably the most critical functions of these signals are to maintain contact during separations of mothers and offspring, as well as to facilitate recognition and reunions within mother-offspring dyads (e.g., [13, 14, 41, 42, 44, 46–48]). Some of the most studied contact calls in toothed whale mother-calf communication are the bottlenose dolphin signature whistle and beluga contact call. Eistla’s production of the type E call in separation and reunion contexts is similar to acoustic behavior that has been shown for wild [25] and captive [13, 14] beluga mothers separated from their calves, as well as bottlenose dolphin mothers that whistle during dyad separations [44, 49–51] and to facilitate reunions [48, 50, 52]. Additionally, characteristics of type E signals were consistent with beluga contact calls as these signals are also known to be distinctive, broadband pulsed or mixed calls that are highly stereotyped and long in duration (> 1 s [13–15, 17, 26]). As previously stated, narwhals and belugas are closely related phylogenetically [53], so it is likely that such similar calls serve similar functions.

Contact call use in odontocete offspring may also be critical in facilitating mother-calf recognition and reunions. For example, Indo-Pacific bottlenose dolphin calves produce signature whistles more reliably during separations than their mothers and appear to be responsible for initiating reunions [46]. Vergara et al. [17] reported vocalization exchanges for a beluga mother-calf dyad in which the production of the mother’s contact call preceded or followed calf pulse trains which are believed to be rudimentary contact calls of beluga calves in the first few months of life [13, 14]. Type E and C signals were produced commonly in call and response vocal exchanges so we speculate that the type C call was the contact call of Eistla’s calf, especially as the characteristics of this call type were also consistent with the contact calls of beluga calves. Akin to call type C, beluga calves produce mixed calls with tremulous whistle components overlapping pulsed components at a similar developmental stage (Eistla’s calf estimated to be < five months old, and a four-month-old beluga calf [14]). Beluga calf contact calls, while clearly stereotyped, do not appear to reach full stereotypy until approximately two years of age [13, 14]. The lack of full stereotypy in type C signals as they appeared on spectrograms (Fig 3) was consistent with the appearance of beluga contact calls during ongoing development [13, 14]. This was also the case with additional sounds which we attributed to Eistla’s calf. These signals were tremulous in nature and appeared similar to beluga calf signals with the same quality of sound production at a similar age [13, 14]. Thus, like what has been described for dolphin calves [32] and belugas [13, 14], narwhal calves may lack the motor-control to produce adult-like vocalizations until they are older.

The social structure of individual cetacean species appears to be intricately tied to the common signals which develop within each species’ communication system (e.g., [54, 55]). Both bottlenose dolphin [56] and beluga societies [57] have fission-fusion characteristics whereby grouping patterns and social group membership are dynamic. Individually distinct contact calls would be a useful adaptation for these species as these signals have the potential to allow for identification among closely associated conspecifics and within separated mother-calf dyads [22, 26, 58]. However, calves of these species appear to develop their contact calls differently. Bottlenose dolphin calves generally develop signature whistles that differ from their mother’s signature contour [47, 59–64], while beluga calves first develop contact calls similar to those of their mothers [13, 14].

Little is known regarding narwhal social structure, although it has been speculated that narwhal societies are “matrifocal” [65, 66]. However, some narwhal herding and clustering patterns (e.g., [67]) share characteristics with mixed-age beluga herds and some smaller beluga social group types [57]. Thus, narwhal societies may have some fission-fusion characteristics which could be reflected in the development of individually distinct calls. If type C was indeed the contact call of Eistla’s calf, then it appears that narwhal calves may develop early contact call types that differ from the types produced by their mothers, an indicator that calves develop calls which distinguish them from animals they are closely associated with [64]. Future research on narwhal social structure, such as studies involving association networks and genetic testing of kinship (e.g., [57]) would be necessary in order to disentangle the role of social structure in calf contact call development.

### Narwhal contact calls: A case for individual vocal signatures?

This is the second study to show that narwhal vocal repertoires have distinct call types that appear to be unique to the signaler’s repertoire. While the call types of the mother and calf reported here differed from each other, from the additional two tagged females in this study, and from the call types reported for two tagged whales from another study (based on Figs 2 and 3 in [10]), it remains unclear whether narwhal contact calls act as individual vocal signatures, despite showing evidence of individual specificity. Many taxa in the animal kingdom have individually distinctive calls, but individual vocal signatures are rarer as this requires a signal to encode the identity of the individual to which the signal belongs [27]. In order to determine whether narwhal calls represent signatures, it must be determined if these signals broadcast identity through testing in the field.

In lieu of methods designed for such purposes (e.g., playback studies for bottlenose dolphins [22–24]), we wanted to explore whether the contact calls presented here acted like other individually specific vocal signatures, so we focused on the acoustic behavior surrounding type E and C production. Types E, C, and T signals were commonly produced in series of the same call type, a characteristic of signature whistles. Janik and colleagues [30] developed the SIGID method to identify the signature whistles of free-ranging bottlenose dolphins which could be conservatively identified if a whistle appeared within 1–10 seconds of another whistle of the same type. We also found that types E, C and T signals were prominently produced within 1–10 seconds of emissions of the same signal type. Surprisingly, type F was not produced in a series in Freya’s acoustic record, but this may have been due to the record length and/or small number of contact calls recorded. Consequently, the SIGID method may have a universal application for investigating possible individual signatures of species exclusively located in extreme habitats, but more empirical studies of the SIGID method in narwhals and on other species would be critical in determining the value of its application. Preliminary evidence has shown that narwhal contact calls are individually distinctive, and we speculate that these calls act as individual signatures given their similar contextual use and structural similarity to other odontocete signature signal types. Future studies are critical in confirming whether these signals continue to exhibit distinctiveness across more individuals and whether these signals are used for broadcasting identity to conspecifics.

## Conclusions

We provide evidence of contact call use in narwhal mother-calf communication as a mother narwhal produced a distinct, highly stereotyped call type when separated from her calf. Additionally, we describe what we believe to be contact call production in a narwhal calf, as we found a second stereotyped call type within the acoustic recordings used in vocal exchanges with the mother’s contact call type. We provide further evidence that narwhal vocal repertoires contain stereotyped, possibly individualized call types used for maintaining contact, but it remains unknown whether these distinct contact call types are individual vocal signatures. Future studies of narwhal mother-calf communication are needed to confirm our speculations about stereotyped call use in calf repertoires and to further elucidate the role of narwhal calf calls in maintaining contact within the mother-calf dyad.

## Supporting information

S1 Audio Example of type E call. (WAV)

S2 Audio Example of type C call. (WAV)

S3 Audio Example of type E and C call and response exchange. (WAV)

S4 Audio Example of Thora’s type T call. (WAV)

S5 Audio Example of Freya’s type F call. (WAV)

S1 Table Distribution of sounds and call types across each female’s record. Column E shows the sum of columns A-D, while column G is the sum of columns E & F.
(PNG)

## References

[1] Heide-Jørgensen MP, Dietz R, Laidre KL, Richard P. Autumn movements, home ranges, and winter density of narwhals (*Monodon monoceros*) tagged in Tremblay Sound, Baffin Island. Polar Biol. 2002; 25: 331–341.

[2] Heide-Jørgensen MP, Laidre KL, Burt ML, Borchers DL, Marques TA, Hansen RG, et al. Abundance of narwhals (*Monodon monoceros*) on the hunting grounds in Greenland. J Mammal. 2010; 91: 1135–1151.

[3] Ford JKB, Fisher HD. Underwater acoustic signals of the narwhal (*Monodon monoceros*). Can J Zool. 1978; 56: 552–560.

[4] Møhl B, Surlykke A, Miller LA. High intensity narwhal clicks. In: Thomas JA, Kastelein RA, editors. Sensory abilities of cetaceans. New York: Plenum Press; 1990. p. 295–303.

[5] Miller LA, Pristed J, Møhl B, Surlykke A. The click-sounds of narwhals (*Monodon monoceros*) in Inglefield Bay, Northwest Greenland. Mar Mamm Sci. 1995; 11: 491–502.

[6] Stafford KM, Laidre KL, Heide-Jørgensen MP. First acoustic recordings of narwhals (*Monodon monoceros*) in winter. Mar Mamm Sci. 2012; 28: E197–E207.

[7] Rasmussen MH, Koblitz JC, Laidre KL. Buzzes and high-frequency clicks recorded from narwhals (*Monodon monoceros*) at their wintering ground. Aquat Mamm. 2015; 41: 256–264.

[8] Blackwell SB, Tervo OM, Conrad AS, Sinding MHS, Hansen RG, Ditlevsen S, et al. Spatial and temporal patterns of sound production in East Greenland narwhals. PLoS ONE. 2018; 13(6): e0198295. doi: 10.1371/journal.pone.0198295 29897955 PMC5999075

[9] Watkins WA, Schevill WE, Ray C. Underwater sounds of *Monodon* (Narwhal). J Acoust Soc Am. 1971; 49: 595–599.

[10] Shapiro AD. Preliminary evidence for signature vocalizations among free-ranging narwhals (*Monodon monoceros*). J Acoust Soc Am. 2006; 120: 1695–1705. doi: 10.1121/1.2226586 17004490

[11] Marcoux M, Auger-Méthé M, Humphries MM. Variability and context specificity of narwhal (*Monodon monoceros*) whistles and pulsed calls. Mar Mamm Sci. 2012; 28: 649–665.

[12] Walmsley SF, Rendell L, Hussey NE, Marcoux M. Vocal sequences in narwhals (*Monodon monoceros*). J Acoust Soc Am. 2020; 147: 1078–1091. doi: 10.1121/10.0000671 32113269

[13] Ames AE, Vergara V. Trajectories of vocal repertoire development in beluga (*Delphinapterus leucas*) calves: insights from studies a decade apart. Aquat Mamm. 2020; 46: 344–366.

[14] Vergara V, Barrett-Lennard LG. Vocal development in a beluga calf (*Delphinapterus leucas*). Aquat Mamm. 2008; 34: 123–143.

[15] Panova E, Agafonov A, Belikov R, Melnikova F. Vocalizations of captive beluga whales, *Delphinapterus leucas*: Additional evidence for contact signature “mixed” calls in belugas. Mar Mamm Sci. 2017; 33: 889–903.

[16] Karlsen J, Bisther A, Lydersen C, Haug T, Kovacs K. Summer vocalisations of adult male white whales (*Delphinapterus leucas*) in Svalbard, Norway. Polar Biol. 2002; 25: 808–817.

[17] Vergara V, Michaud R, Barrett-Lennard L. What can captive whales tell us about their wild counterparts? Identification, usage, and ontogeny of contact calls in belugas (*Delphinapterus leucas*). Int J Comp Psychol. 2010; 23: 278–309.

[18] Hay KA, Mansfield AW. Narwhal, *Monodon monoceros* Linnaeus, 1758. In: Ridgway SH, Richardson RJ, editors. Handbook of marine mammals, vol 4. London: Academic Press; 1989. p. 145–176.

[19] Caldwell MC, Caldwell DK. Individualized whistle contours in bottle-nosed dolphins (*Tursiops truncatus*). Nature. 1965; 207: 434–435.

[20] Caldwell MC, Caldwell DK, Tyack PL. Review of the signature-whistle hypothesis for the Atlantic bottlenose dolphin. In: Leatherwood S, Reeves RR, editors. The bottlenose dolphin. New York: Academic Press; 1990. p. 199–234.

[21] Janik VM, Sayigh LS. Communication in bottlenose dolphins: 50 years of signature whistle research. J Comp Physiol A. 2013; 199: 479–489. doi: 10.1007/s00359-013-0817-7 23649908

[22] Sayigh LS, Tyack PL, Wells RS, Solow AR, Scott MD, Irvine AB. Individual recognition in wild bottlenose dolphins: a field test using playback experiments. Animal Behav. 1999; 57: 41–50. doi: 10.1006/anbe.1998.0961 10053070

[23] Janik VM, Sayigh LS, Wells RS. Signature whistle shape conveys identity information to bottlenose dolphins. Proc Natl Acad Sci. 2006; 103: 8293–8297. doi: 10.1073/pnas.0509918103 16698937 PMC1472465

[24] Sayigh LS, Wells RS, Janik VM. What’s in a voice? Dolphins do not use voice cues for individual recognition. Anim Cogn. 2017; 20: 1067–1079. doi: 10.1007/s10071-017-1123-5 28791513 PMC5640738

[25] Van Parijs SM, Lydersen C, Kovacs KM. Sounds produced by individual white whales, *Delphinapterus leucas*, from Svalbard during capture. J Acoust Soc Am. 2003; 113: 57–60. doi: 10.1121/1.1528931 12558246

[26] Vergara V, Mikus MA. Contact call diversity in natural beluga entrapments in an Arctic estuary: Preliminary evidence of vocal signatures in wild belugas. Mar Mamm Sci. 2019; 35: 434–465.

[27] Boughman JW, Moss CF. Social sounds: vocal learning and development of mammal and bird calls. In: Simmons AM, Popper AN, Fay RR, editors. Acoustic communication. New York: Springer; 2003. p. 138–224.

[28] Heide‐Jørgensen MP, Blackwell SB, Williams TM, Sinding MH, Skovrind M, Tervo OM, et al. Some like it cold: Temperature‐dependent habitat selection by narwhals. Ecol Evol. 2020; 10: 8073–8090. doi: 10.1002/ece3.6464 32788962 PMC7417212

[29] Heide-Jørgensen MP, Nielsen NH, Hansen RG, Schmidt HC, Blackwell SB, Jørgensen OA. The predictable narwhal: satellite tracking shows behavioural similarities between isolated subpopulations. J Zool. 2015; 297: 54–65.

[30] Janik VM, King SL, Sayigh LS, Wells RS. Identifying signature whistles from recordings of groups of unrestrained bottlenose dolphins (*Tursiops truncatus*). Mar Mamm Sci. 2013; 29: 109–122.

[31] Reiss D. Observations on the development of echolocation in young bottlenose dolphins. In: Nachtigall PE, Moore PWB, editors. Animal sonar. Boston: Springer; 1988. p. 121–127.

[32] Killebrew D, Mercado E, Herman L, Pack A. Sound production of a neonate bottlenose dolphin. Aquat Mamm. 2001; 27: 34–44.

[33] Ridgway SH, Carder DA, Kamolnick T, Smith RR, Schlundt CE, Elsberry WR. Hearing and whistling in the deep sea: depth influences whistle spectra but does not attenuate hearing by white whales (*Delphinapterus leucas*)(Odontoceti, Cetacea). J Exp Biol. 2001; 204: 3829–3841. 11807101 10.1242/jeb.204.22.3829

[34] Tervo OM, Ditlevsen S, Ngô MC, Nielsen NH, Blackwell SB, Williams TM, et al. Hunting by the stroke: how foraging drives diving behavior and locomotion of East-Greenland narwhals (Monodon monoceros). Front Mar Sci. 2020; 7:596469.

[35] Johnson M. On-animal methods for studying echolocation in free-ranging animals. In: Surlykke A, Nachtigall PE, Fay RR, Popper AN, editors. Biosonar. Springer Handbook of Auditory Research, vol 51. Springer, New York, NY. 2014. p. 195–229.

[36] Miller PJ. Mixed-directionality of killer whale stereotyped calls: A direction of movement cue? Behav Ecol Sociobiol. 2002; 52: 262–270.

[37] Lammers MO, Au WW. Directionality in the whistles of Hawaiian spinner dolphins (*Stenella longirostris*): A signal feature to cue direction of movement? Mar Mamm Sci. 2003; 19: 249–264.

[38] Branstetter BK, Black A, Bakhtiari K. Discrimination of mixed-directional whistles by a bottlenose dolphin (*Tursiops truncatus*). J Acoust Soc Am. 2013; 134: 2274–2285. doi: 10.1121/1.4816404 23967957

[39] Whitehead H. Babysitting, dive synchrony, and indications of alloparental care in sperm whales. Behav Ecol Sociobiol. 1996; 38: 237–244.

[40] Jouventin P, Aubin T, Lengagne T. Finding a parent in a king penguin colony: the acoustic system of individual recognition. Anim Behav. 1999; 57: 1175–1183. doi: 10.1006/anbe.1999.1086 10373249

[41] Trillmich F. Mutual mother-pup recognition in Galapagos fur seals and sea lions: cues used and functional significance. Behaviour. 1981; 78: 21–42.

[42] Insley SJ. Mother–offspring vocal recognition in northern fur seals is mutual but asymmetrical. Anim Behav. 2001; 61: 129–137. doi: 10.1006/anbe.2000.1569 11170703

[43] Weiß BM, Ladich F, Spong P, Symonds H. Vocal behavior of resident killer whale matrilines with newborn calves: The role of family signatures. J Acoust Soc Am. 2006; 119: 627–635. doi: 10.1121/1.2130934 16454316

[44] Sayigh LS, Tyack PL, Wells RS, Scott MD. Signature whistles of free-ranging bottlenose dolphins *Tursiops truncatus*: stability and mother-offspring comparisons. Behav Ecol Sociobiol. 1990; 26: 247–260.

[45] Gridley T, Cockcroft VG, Hawkins ER, Blewitt ML, Morisaka T, Janik VM. Signature whistles in free‐ranging populations of Indo‐Pacific bottlenose dolphins, *Tursiops aduncus*. Mar Mamm Sci. 2014; 30: 512–527.

[46] Smolker RA, Mann J, Smuts BB. Use of signature whistles during separations and reunions by wild bottlenose dolphin mothers and infants. Behav Ecol Sociobiol. 1993; 33: 393–402.

[47] Tyack PL, Sayigh LS. Vocal learning in cetaceans. In: Snowdon CT, Hausberger M, editors. Social influences on vocal development. Cambridge: Cambridge University Press; 1997. p. 208–233.

[48] King SL, Guarino E, Keaton L, Erb L, Jaakkola K. Maternal signature whistle use aids mother-calf reunions in a bottlenose dolphin, *Tursiops truncatus*. Behav Processes. 2016; 126: 64–70. doi: 10.1016/j.beproc.2016.03.005 26992371

[49] Sayigh LS, Tyack PL, Wells RS, Scott MD, Irvine AB. Sex difference in signature whistle production of free-ranging bottlenose dolphins, *Tursiops truncates*. Behav Ecol Sociobiol. 1995; 36: 171–177.

[50] Mello I, Amundin M. Whistle production pre-and post-partum in bottlenose dolphins (*Tursiops truncatus*) in human care. Aquat Mamm. 2005; 31: 169–175.

[51] King SL, Sayigh LS, Wells RS, Fellner W, Janik VM. Vocal copying of individually distinctive signature whistles in bottlenose dolphins. Proc Royal Soc B. 2013; 280: 20130053. 10.1098/rspb.2013.0053 PMC3619487 23427174

[52] Kuczaj SA II, Eskelinen HC, Jones BL, Borger-Turner JL. Gotta go, mom’s calling: Dolphin (*Tursiops truncatus*) mothers use individually distinctive acoustic signals to call their calves. Anim Behav Cogn. 2015; 2: 88–95.

[53] Messenger SL, McGuire JA. Morphology, molecules, and the phylogenetics of cetaceans. Syst Biol. 1998; 47: 90–124. doi: 10.1080/106351598261058 12064244

[54] Tyack PL. Acoustic communication under the sea. In: Hopp SL, Owren MJ, Evans CS, editors. Animal acoustic communication. Berlin: Springer; 1998. p. 163–220.

[55] Tyack PL. Convergence of calls as animals form social bonds, active compensation for noisy communication channels, and the evolution of vocal learning in mammals. J Comp Psychol. 2008; 122: 319–331. doi: 10.1037/a0013087 18729661

[56] Connor RC, Wells RS, Mann J, Read AJ. Social relationships in a fission-fusion society. In: Mann J, Connor RC, Tyack PL, Whitehead H, editors. Cetacean societies: Field studies of whales and dolphins. Chicago: University of Chicago Press; 2000. p. 91–126.

[57] O’Corry-Crowe G, Suydam R, Quakenbush L, Smith TG, Lydersen C, Kovacs KM, et al. Group structure and kinship in beluga whale societies. Sci Rep. 2020; 10: 1–21. doi: 10.1038/s41598-019-56847-4 32651398 PMC7351962

[58] Tyack PL. Dolphins communicate about individual-specific social relationships. In: de Waal FBM, Tyack PL, editors. Animal social complexity: Intelligence, culture, and individualized societies. Cambridge: Harvard University Press; 2003. p. 342–361.

[59] Caldwell MC, Caldwell DK. The whistle of the Atlantic bottlenosed dolphin (*Tursiops truncatus*)—ontogeny. In: Winn HE, Olla BL, editors. Behavior of marine animals, vol 3, cetaceans. New York: Plenum Press; 1979. p. 369–401.

[60] Sayigh LS. Development and functions of signature whistles of free-ranging bottlenose dolphins, Tursiops truncatus. Ph.D. Thesis, Massachusetts Institute of Technology and Woods Hole Oceanographic Institution. 1992. Available from: https://apps.dtic.mil/dtic/tr/fulltext/u2/a259362.pdf

[61] Tyack PL. Development and social functions of signature whistles in bottlenose dolphins *Tursiops truncatus*. Bioacoustics. 1997; 8: 21–46.

[62] Bojanowski E, Veit F, Todt D. The development of a bivocal signature whistle in a bottlenose dolphin calf. Eur Res Cetaceans. 2000; 14: 70–74.

[63] Miksis JL, Tyack PL, Buck JR. Captive dolphins, *Tursiops truncatus*, develop signature whistles that match acoustic features of human-made model sounds. J Acoust Soc Am. 2002; 112: 728–739. doi: 10.1121/1.1496079 12186052

[64] Fripp D, Owen C, Quintana-Rizzo E, Shapiro A, Buckstaff K, Jankowski K, et al. Bottlenose dolphin (*Tursiops truncatus*) calves appear to model their signature whistles on the signature whistles of community members. Anim Cogn. 2005; 8: 17–26. doi: 10.1007/s10071-004-0225-z 15221637

[65] Palsbøll P, Heide-Jørgensen MP, Dietz R. Distribution of mtDNA haplotypes in narwhals, *Monodon monoceros*. Heredity. 1997; 78: 284–292. doi: 10.1038/hdy.1997.43 9119704

[66] Whitehead H. Cultural selection and genetic diversity in matrilineal whales. Science. 1998; 282: 1708–1711. doi: 10.1126/science.282.5394.1708 9831562

[67] Marcoux M, Auger-Méthé M, Humphries MM. Encounter frequencies and grouping patterns of narwhals in Koluktoo Bay, Baffin Island. Polar Biol. 2009; 32: 1705–1716.

