# Supplementary figures and images for: Evidence of stereotyped contact call use in narwhal (Monodon monoceros) mother-calf communication
Source: PLoS One. 2021 Aug 27;16(8):e0254393. doi: 10.1371/journal.pone.0254393 (PMC8396719; doi:10.1371/journal.pone.0254393)

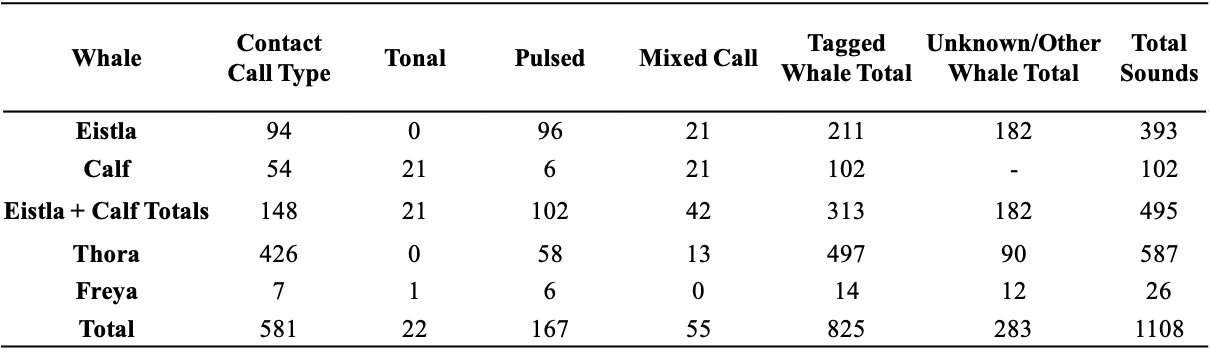

Supplement: S1 Table — Column E shows the sum of columns A-D, while column G is the sum of columns E & F. (PNG) [file pone.0254393.s006.png]
